# Organic Phosphorus in Nervous Diseases

**Published:** 1907-04

**Authors:** 


### Organic Phosphorus in Nervous Diseases.

Dr. Alfred Gordon, Associate in Nervous and Mental Diseases, Jefferson Medical College, Philadelphia, writes on the role of organic phosphorus (*Medicine,* December, 1906,) in the asthenia of nervous affections. Phosphorus exists in the brain and nerves in glycerophosphoric combination and is decomposed into inorganic compounds under mental activity, sexual excitement, and the like. Exhaustive disorders of the nervous system should be treated with organic forms of phosphorus, to restore the phosphatic loss, as inorganic forms of phosphorus are not absorbed.

Gordon used the glycerophosphates, which appear to give the best results, in 56 cases of syphilitic nervous affections, neurasthenia and insanity. In the specific cases the glycerophosphates showed the most beneficial effect on the asthenia and reenforced the mecurials and iodides, the special symptoms improving. In the neurasthenias, some of which were post-grippal, as well as in the other cases the favorable effect was pronounced. Improvement only began when the glycerophosphates were adminsstered and ceased when they were withdrawn.

The author concludes that the glycerophosphates effected an increase in weight, improvement in strength and mental activity, and a sensation of well being. They are rapidly and completely assimilated, acting as regenerators and stimulants to nutrition and metabolism.

